# The Sodium-Glucose Co-Transporter 2 Inhibitor Empagliflozin Improves Diabetes-Induced Vascular Dysfunction in the Streptozotocin Diabetes Rat Model by Interfering with Oxidative Stress and Glucotoxicity

**DOI:** 10.1371/journal.pone.0112394

**Published:** 2014-11-17

**Authors:** Matthias Oelze, Swenja Kröller-Schön, Philipp Welschof, Thomas Jansen, Michael Hausding, Yuliya Mikhed, Paul Stamm, Michael Mader, Elena Zinßius, Saule Agdauletova, Anna Gottschlich, Sebastian Steven, Eberhard Schulz, Serge P. Bottari, Eric Mayoux, Thomas Münzel, Andreas Daiber

**Affiliations:** 1 2nd Medical Clinic, Department of Cardiology, Medical Center of the Johannes Gutenberg University, Mainz, Germany; 2 Laboratory of Fundamental and Applied, Bioenergetics, INSERM U1055, Grenoble-Alpes Université et Pôle de Biologie, CHU, Grenoble, France; 3 Boehringer Ingelheim Pharma GmbH & Co. KG, Ingelheim, Germany; University of Padova, Italy

## Abstract

**Objective:**

In diabetes, vascular dysfunction is characterized by impaired endothelial function due to increased oxidative stress. Empagliflozin, as a selective sodium-glucose co-transporter 2 inhibitor (SGLT2i), offers a novel approach for the treatment of type 2 diabetes by enhancing urinary glucose excretion. The aim of the present study was to test whether treatment with empagliflozin improves endothelial dysfunction in type I diabetic rats via reduction of glucotoxicity and associated vascular oxidative stress.

**Methods:**

Type I diabetes in Wistar rats was induced by an intravenous injection of streptozotocin (60 mg/kg). One week after injection empagliflozin (10 and 30 mg/kg/d) was administered via drinking water for 7 weeks. Vascular function was assessed by isometric tension recording, oxidative stress parameters by chemiluminescence and fluorescence techniques, protein expression by Western blot, mRNA expression by RT-PCR, and islet function by insulin ELISA in serum and immunohistochemical staining of pancreatic tissue. Advanced glycation end products (AGE) signaling was assessed by dot blot analysis and mRNA expression of the AGE-receptor (RAGE).

**Results:**

Treatment with empagliflozin reduced blood glucose levels, normalized endothelial function (aortic rings) and reduced oxidative stress in aortic vessels (dihydroethidium staining) and in blood (phorbol ester/zymosan A-stimulated chemiluminescence) of diabetic rats. Additionally, the pro-inflammatory phenotype and glucotoxicity (AGE/RAGE signaling) in diabetic animals was reversed by SGLT2i therapy.

**Conclusions:**

Empagliflozin improves hyperglycemia and prevents the development of endothelial dysfunction, reduces oxidative stress and improves the metabolic situation in type 1 diabetic rats. These preclinical observations illustrate the therapeutic potential of this new class of antidiabetic drugs.

## Introduction

Diabetes mellitus is one of the major risk factors for the development of cardiovascular disease [1]. Several studies have demonstrated that endothelial dysfunction due to increased oxidative stress is frequently encountered in the diabetic state (for review see [2]). The vascular NADPH oxidase and an uncoupled endothelial nitric oxide synthase (eNOS, type 3) have been identified as enzymatic sources of increased vascular production of reactive oxygen species (ROS) [3]. Pharmacological treatment of diabetic animals with HMG-CoA-reductase inhibitors, AT_1_-receptor blockers or heme oxygenase-1 induction by NO donor therapy with pentaerithrityl tetranitrate have been demonstrated to prevent the activation of the NADPH oxidase and to recouple the dysfunctional eNOS [4]–[6]. The mechanisms underlying eNOS uncoupling in vessels from diabetic animals include increased functional depletion of BH_4_ due to the oxidation to the ^•^BH_3_ radical, oxidation of the zinc-sulfur-complex and S-glutathionylation of the enzyme [4], [6]. Adverse phosphorylation of eNOS at Thr495 and Tyr657 may potentially play a significant role as well [7], [8]. Another major concept of diabetic pathology is based on direct glucotoxicity, including increased formation of advanced glycation end products (AGE) and their signaling via specific receptors (RAGE) leading to vascular dysfunction and end organ damage [9], [10]. Most importantly, oxidative stress and AGE/RAGE components interact with each other in a cross-talk fashion, wherein AGE/RAGE signaling can activate sources of reactive oxygen species (ROS) [11], [12] and normalization of mitochondrial ROS formation in turn normalizes hyperglycemic damage by decreasing AGE/RAGE signaling [13]. In addition, increased vascular oxidative stress can lead to immune cell activation [14], [15] or is even mediated by inflammatory cells, as was recently demonstrated in the angiotensin II infusion model [16].

A new class of anti-diabetic drugs targets the sodium-glucose co-transporter 2 (SGLT2), which is the main glucose transporter of the kidney, located in the S1 and S2 segments of the proximal tubule and is responsible for the reabsorption of >90% of the glucose from primary urine [17]. SGLT2 inhibition (SGLT2i) reduces the reabsorption of glucose and therefore enhances urinary glucose excretion, consequently decreasing both fasting and postprandial hyperglycemia. Since conventional anti-diabetic therapies rely on insulin secretion, their efficacy may get lost over time due to progressing β-cell dysfunction and desensitization to insulin signaling (especially with increasing age) [18]. Therapy with SGLT2i does not share these drawbacks, since its action is independent of insulin secretion and signaling. Likewise, in contrast to conventional anti-diabetics, SGLT2i removes excessive glucose from the body and thereby prevents glucotoxicity, which should represent a straight-forward strategy to prevent hyperglycemia-induced damage. Empagliflozin is a SGLT2i that was recently approved for clinical use in the United States of America and Europe. According to randomized, placebo-controlled, double-blind clinical studies, empagliflozin had a good safety and tolerability profile in healthy Japanese male subjects [19], [20].

With the present study we sought to test whether treatment of diabetic animals with the SGLT2i empagliflozin improves endothelial dysfunction, oxidative stress, AGE/RAGE signaling and inflammation in a well-characterized rat model of type 1 diabetes mellitus [3].

## Materials and Methods

### Materials

The High-Capacity cDNA Reverse Transcription Kit was purchased from Applied Biosystems, Darmstadt, Germany. All oligonucleotides and dual labeled probes were purchased from MWG Biotech, Ebersberg, Germany. The Bradford reagent was obtained from BioRad, Munich, Germany. For isometric tension studies, nitroglycerin (GTN) was used from a Nitrolingual infusion solution (1 mg/ml) from G.Pohl-Boskamp (Hohenlockstedt, Germany). SGLT2-inhibitor (SGLT2i, empagliflozin) was a kind gift from Boehringer Ingelheim Pharma GmbH & Co KG (Biberach, Germany). For induction of diabetes we used streptozotocin (STZ) from Fluka (Steinheim, Germany). L-012 (8-amino-5-chloro-7-phenylpyrido[3,4-d]pyridazine-1,4-(2H,3H)dione sodium salt) was purchased from Wako Pure Chemical Industries (Osaka, Japan). All other chemicals were of analytical grade and were obtained from Sigma-Aldrich, Fluka or Merck.

### Animals and in vivo treatment

All animals were treated in accordance with the Guide for the Care and Use of Laboratory Animals as adopted by the U.S. National Institutes of Health and approval was granted by the Ethics Committee of the University Hospital Mainz and the Landesuntersuchungsamt Rheinland-Pfalz (Koblenz, Germany; permit number: 23 177-07/G 12-1-025). All surgery was performed under isoflurane or ketamine/xylazine anesthesia, and all efforts were made to minimize suffering. A total number of 48 male Wistar Rats (6 weeks old, 300 g, Charles River Laboratories, Sulzfeld, Germany) were divided into 4 treatment groups: untreated controls (Ctr), streptozotocin-induced diabetes mellitus type 1 with placebo (STZ), SGLT2i low dose (10 mg/kg/d p.o.) or SGLT2i high dose (30 mg/kg/d p.o.) therapy. Given the maximal acute glucosuric effect of empagliflozin, the dose of 10 mg/kg/d was chosen according to previous animal studies [21]–[23]. Moreover, considering the higher metabolism of rats, the dose of 30 mg/kg/d allowed a higher 24 h drug exposure as already observed in previous study with chronic treatment [24]. The study was performed as 3 sub-studies with 4, 5 and 3 animals per group. Not all animals were used for all assays. For induction of diabetes mellitus type 1, rats were injected with a single dose of STZ into the vena dorsalis penis (60 mg/kg s.c., in 5 mM pH 4.5 citrate buffer). Control animals were injected with the solvent. SGLT2i treatment by drinking water, containing the requested concentrations of the drug to reach the desired doses of 10 and 30 mg/kg/d, was started 1 week after STZ-injection and continued for 7 weeks. After 8 weeks of total treatment duration, animals were killed under isoflurane anesthesia by transection of the diaphragm and removal of the heart and thoracic aorta. For protein and mRNA expression also the abdominal part and the arch of the aorta were used as well. Diabetes was diagnosed by measuring glucose levels and glycosylated hemoglobin (HbA1c) in whole blood (for STZ-treated rats it was diluted 1∶5 with NaCl solution) using the ACCU-CHEK Sensor system from Roche Diagnostics GmbH (Mannheim, Germany) and A1C Now^+^ system from Bayer HealthCare Diabetes Care (Basel, Switzerland), respectively. STZ treatment was previously shown to be a valid type 1 diabetes model and hyperglycemic complications such as vascular dysfunction and oxidative stress were completely reversed by insulin administration [25].

### Detection of serum cholesterol, triglyceride and interferon-γ levels

Serum cholesterol, triglyceride and interferon-γ levels were analysed in the Department of Clinical Chemistry, University Hospital Mainz, Germany, using the daily routine facilities for in-patient care. Total cholesterol in serum was also assessed by HF5 (Superon GmbH, Dernbach, Germany). More detailed information on determination of cholesterol by Field-Flow Fractionation (FFF) can be found in the online supplemental data in File S1.

### Immunohistochemistry of pancreatic tissue

Pancreatic segments were fixed in paraformaldehyde (4%), paraffin-embedded and stained with primary antibodies against glucagon (1∶4000, Abcam, UK) and insulin (1∶200, LifeSpan BioSciences, Seattle, USA); depending upon the species of primary antibodies, appropriate biotinylated secondary antibodies were used at dilutions according to the manufacturer's instructions. For immunochemical detection ABC reagent (Vector) and then DAB reagent (peroxidase substrate Kit, Vector) were used as substrates.

### Detection of insulin serum levels by ELISA and by RIA

Venous blood was transferred into serum-vials, left on ice for 30 min and centrifuged for 10 min at 2000 g. The supernatant (serum) was stored at −80°C. Quantitative measurement of serum insulin levels was performed using a rat insulin ELISA (DRG Instruments GmbH, Marburg, Germany) and a rat insulin RIA (Cat. # SRI-13K, Millipore, St. Charles, Missouri, USA) according to the manufacturer's instructions.

### Histological staining of aortic rings and pancreatic tissue

Sirius red staining for vascular fibrosis and trichrom staining was performed with paraffin-embedded samples of aortic tissue upon deparaffination [26]. The nuclei were prestained with hemalum prior to staining for 1 hour in 0.1% with Sirius red solution in saturated picric acid (1.2%) [26]. Finally, tissue samples were dehydrated with 70%, 96% and 100% isopropanol and coverslipped with Entellan. Trichrom staining (according to Goldner et al.) was performed with paraffin-embedded samples of pancreatic tissue upon deparaffination [26]. After prestaining of the nuclei with haematoxylin (according to Harris et al.), the samples were stained for 5 minutes with mallory red in 100% acetic acid, fuchsin acid and Orange G (Merck, Darmstadt, Germany), followed by 15 minutes in 1% molybdatophosphoric acid hydrate (VWR, Darmstadt, Germany) and 5 minutes in acid light green. Finally tissue samples were dehydrated in glacial acetic acid and 100% ethanol and coverslipped in Entellan.

### Isometric tension studies

Vasodilator responses to acetylcholine (ACh) and nitroglycerin (GTN) and vasoconstrictor responses to KCl were assessed with endothelium-intact isolated rat aortic rings mounted for isometric tension recordings in organ chambers, preconstricted with phenylephrine (PheE), as described previously [5], [27].

### Detection of oxidative stress in whole blood, cardiac membrane fractions and aorta

Whole blood leukocyte-dependent ROS formation was measured in fresh blood (in citrate tubes) which was diluted 1∶50 in PBS with Ca^2+^/Mg^2+^ (each 1 mM) and L-012. The oxidative burst (activation of the phagocytic NADPH oxidase) was triggered by the phorbol ester PDBu (10 µM) or the fungal endotoxin zymosan A (50 µg/ml) and assessed with L-012 (100 µM) enhanced chemiluminescence (ECL) [28]. NADPH oxidase activity in cardiac membrane fractions was determined by lucigenin (5 µM) ECL in the presence of NADPH (200 µM) using a Lumat LB9507 single photon counter as described [5], [29]. Vascular ROS formation was determined using dihydroethidium (DHE, 1 µM)-dependent fluorescence microtopography in aortic cryo-sections as described [5], [29]. To investigate involvement of eNOS uncoupling in ROS production and endothelial dysfunction within the endothelial monolayer, aortic rings were preincubated with the NOS inhibitor L-NAME [5], [25], [29]. ROS-derived red fluorescence was detected using a Zeiss Axiovert 40 CFL microscope, Zeiss lenses and Axiocam MRm camera. Intensity of the DHE oxidation products' fluorescence was evaluated by densitometry.

### Western blot and dot blot analysis

Protein expression and modification was assessed by standard Western and dot blot analysis using established protocols [5], [25], [29]. Isolated aortic tissue was frozen and homogenized in liquid nitrogen. Proteins were separated by SDS-Page and blotted onto nitrocellulose membranes. After blocking, immunoblotting was performed with the following antibodies: monoclonal mouse α-actinin (100 kDa) as a control for loading and transfer, polyclonal rabbit NADPH oxidase isoform 1 (Nox1) (1∶500, Abcam, UK) and monoclonal mouse NADPH oxidase isoform 2 (Nox2) (gp91^phox^, 1∶1000, BD Biosciences, USA), monoclonal mouse eNOS (1∶1000, BD Biosciences, USA), polyclonal rabbit phospho-Ser1177-eNOS (1∶1000, Upstate Biotechnology, MA, USA), monoclonal mouse dihydrofolate reductase (1 µg/ml, Abnova Corp., Germany), monoclonal mouse heme oxygenase-1 (HO-1) (4 µg/ml, Stressgen, San Diego, CA), polyclonal goat cGMP-dependent protein kinase (cGK-I, 1∶200, Santa Cruz Biotechnologies, USA), vasodilator stimulated phosphoprotein (VASP) phosphorylated on serine239 (P-VASP, clone 16C2, 1.5 µg/ml, Calbiochem, UK), polyclonal rabbit monocyte- chemoattractant-protein-1 (MCP-1 or CCL-2, Serotec, UK), polyclonal rabbit receptor for advanced glycation end products (RAGE, 1∶1000, Cell Signaling Technology, Danvers, MA). Detection and quantification were performed by ECL with peroxidase conjugated anti–rabbit/mouse (1∶10000, Vector Lab., Burlingame, CA) and anti-goat (1∶5000, Santa Cruz Biotechnologies, USA) secondary antibodies. Densitometric quantification of antibody-specific bands was performed with a ChemiLux Imager (CsX-1400 M, Intas, Göttingen, Germany) and Gel-Pro Analyzer software (Media Cybernetics, Bethesda, MD).

Advanced glycation end products (AGE) positive proteins were assessed by dot blot analysis of aortic protein homogenate, which was transferred to a Protran BA85 (0.45 µm) nitrocellulose membrane (Schleicher & Schuell, Dassel, Germany) by a Minifold I vacuum dot-blot system (Schleicher&Schuell, Dassel, Germany). Each slot was washed with 250 µl PBS and the membrane was dried for 15 min at 60°C. For detection of AGE's, a mouse monoclonal AGE antibody (Antibodies-online GmbH, Aachen, Germany) was used at a dilution of 1∶1000. Positive dots were detected by enhanced chemiluminescence after incubation with a peroxidase-coupled secondary antibody (1∶10000, Vector Lab., Burlingame, CA). All incubation and washing steps were performed according to the manufacturer's instructions. Densitometric quantification of the dots was performed as described in the Western blot section.

### Detection of serum methylglyoxal levels and liver ALDH-2 activity by HPLC-based quantification

Methylglyoxal serum levels were quantified by an HPLC-based method upon derivatization of the reactive aldehyde as adapted from a previously described protocol [30]. Briefly, 500 µl serum was filtrated by size exclusion centrifugation in a 30 kDa filter device (Millipore, Billierica, MA, USA) for 60 min at 17000 g. The eluate (350 µl) was mixed with 70 µl 7M HClO_4_, 10 µl 100 µM 2,3-dimethylquinoxaline (internal standard) and 70 µl 9.2 mM 1,2-diaminobenzene (derivatization reagent) for 30 min at 25°C. 50 µl of each sample were subjected to HPLC analysis. The system consisted of a Jasco HPLC system and a C_18_-Nucleosil 125×4 100-3 reversed phase column from Macherey & Nagel (Düren, Germany). The mobile phase consisted of solvent A (50 mM citric acid pH 2.2) and solvent B (acetonitrile (90 v/v%) and water (10 v/v%)). The substrate and its products were eluted at a flow rate of 1 ml/min using a gradient (0 min: 37% B; 12 min: 60% B; 12.5 min: 90% B; 13 min: 37% B; 15 min: 37% B), detected by their absorption at 315 nm, and quantified using internal and external standards (methylglyoxal derivatization product served as external standard and 2,3-dimethylquinoxaline was the internal standard). The typical retention times were 3.9 and 4.4 min, respectively.

The activity of ALDH-2 in isolated mitochondria was determined by measuring the conversion of 2-hydroxy-3-nitro-benzaldehyde to 2-hydroxy-3-nitro-benzoic acid as described previously [31]. Briefly, liver samples were homogenized in HEPES buffer (composition in mM: 50 HEPES, 70 sucrose, 220 mannitol, 1 EGTA and 0.033 BSA) and centrifuged at 1500 g (10 min at 4°C) and 2000 g for 5 min (the pellets were discarded). The supernatant was then centrifuged at 20000 g for 20 min, and the pellet resuspended in 1 ml of HEPES buffer. The latter step was repeated and the pellet resuspended in 1 ml of Tris buffer (composition in mM: 10 Tris, 340 sucrose, 100 KCl and 1 EDTA). The mitochondrial fraction (total protein approximately 5–10 mg/ml) was kept on ice and diluted to approximately 1 mg/ml protein in 0.25 ml of PBS and preincubated for 10 min at room temperature. For measurement of ALDH-2 dehydrogenase activity, 2-hydroxy-3-nitro-benzaldehyde (100 µM) was added and the samples were incubated for another 30 min at 37°C. Samples were sonicated, centrifuged at 20000 g (4°C) for 20 min, and the supernatant was purified by size exclusion centrifugation in a 10 kDa filter device (Millipore). 50 µl of each sample were subjected to HPLC analysis. The system consisted of a Jasco HPLC system and a C_18_-Nucleosil 125×4 100-3 reversed phase column from Macherey & Nagel (Düren, Germany). The mobile phase consisted of solvent A (50 mM citric acid pH 2.2) and solvent B (acetonitrile (90 v/v%) and water (10 v/v%)). The substrate and its products were eluted at a flow rate of 1 ml/min using a gradient (0 min: 25% B; 7 min: 50% B; 8 min: 100% B; 9 min: 25% B; 11 min: 25% B), detected by their absorption at 360 nm, and quantified using internal and external standards (2-hydroxy-3-nitro-benzoic acid and 2-hydroxy-3-nitro-benzaldehyde). The typical retention times were 2.4 and 3.7 min, respectively.

### Quantitative reverse transcription real-time PCR (qRT-PCR)

mRNA expression was analyzed with quantitative reverse transcription real-time PCR (qRT-PCR) as previously described [32]. Briefly, total RNA from rat aorta was isolated (RNeasy Fibrous Tissue Mini Kit; Qiagen, Hilden, Germany), and 50 ng of total RNA was used for real-time RT-PCR analysis with the QuantiTect Probe RT-PCR kit (Qiagen). TaqMan Gene Expression assays for monocyte chemoattractant protein-1 (MCP-1 or CCL-2), monocyte/macrophage-specific protein CD68, cytokine interleukin-6 (IL-6), immune-signaling proteins interferon-γ (IFN-γ) and tumor necrosis factor-α (TNF-α), intercellular adhesion molecule-1 (ICAM-1), cyclooxygenase-2 (COX-2) and the receptor for advanced glycation end products (RAGE) as well as TBP were purchased as probe-and-primer sets (Applied Biosystems, Foster City, CA). The comparative Ct method was used for relative mRNA quantification. Gene expression was normalized to the endogenous control, TATA box binding protein (TBP) mRNA, and the amount of target gene mRNA expression in each sample was expressed relative to the control.

### Statistical analysis

Results are expressed as the means ±SEM. Two-way ANOVA (with Bonferroni's correction for comparison of multiple means) was used for comparisons of concentration-relaxation curves. In addition, one-way ANOVA (with Bonferroni's or Dunn's correction for comparison of multiple means) was used for comparisons of vasodilator potency (EC_50_, pD_2_ see Table 1) and efficacy (max. relaxation see Table 1). One-way ANOVA (with Bonferroni's or Dunn's correction for comparison of multiple means) was used for comparisons of weight gain, blood glucose, HbA1c levels, other serum parameters, such as insulin, triglycerides and methylglyoxal levels, histological data, aortic and cardiac ROS formation, protein and mRNA expression, vasoconstriction and hepatic ALDH-2 activity. p values <0.05 were considered as statistically significant. The number of replicates in the different assays may vary due to the death of some rats in the different sub-studies and since some values were excluded when they exceeded a deviation of 2xSD from the means.

**Table 1:** Potencies and efficacies of the endothelium-dependent vasodilator acetylcholine and the endothelium-independent vasodilator nitroglycerin in rat aorta.

	In vivo treatment group			
Parameter^a^	Ctr	STZ	STZ+low SGLT2i	STZ+high SGLT2i
**Potency (pD2), ACh**	7.52±0.07 (n = 12)	7.12±0.06 (n = 9)*	7.33±0.06 (n = 10)^#^	7.28±0.05 (n = 9)* ^#^
**Efficacy (%), ACh**	90.3±1.4 (n = 12)	80.3±2.3 (n = 9)*	91.5±0.7 (n = 10)^#^	85.5±2.4 (n = 9)^§^
**Potency (pD2), GTN**	7.81±0.07 (n = 12)	7.62±0.11 (n = 9)*	7.73±0.08 (n = 10)	7.70±0.12 (n = 9)
**Efficacy (%), GTN**	99.4±0.2 (n = 12)	96.7±1.6 (n = 9)*	99.2±0.1 (n = 10)	98.8±0.3 (n = 9)

a Potency is –log EC_50_ and EC_50_ is the concentration that induces half-maximal relaxation with respect to the maximal relaxation achieved with the highest concentration of the vasodilator used. Efficacy is defined as maximal relaxation obtained with the highest concentration of the vasodilator used. The data are the means ± SEM of the indicated number of animals/group.

*, p<0.05 vs. control and ^#^, p<0.05 vs. STZ-injected and ^§^, p<0.05 vs. low dose SGLT2i treated.

## Results

### Diabetes-related parameters (Table 2)

**Figure 1:**
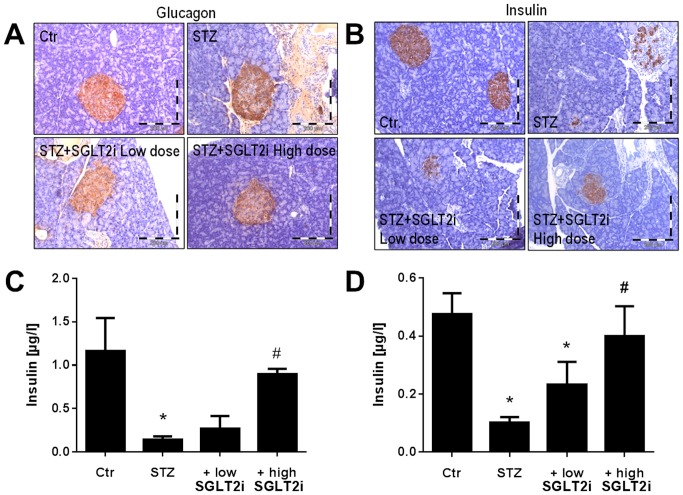
Glucagon and insulin measurements in controls and diabetic rats 8 weeks after STZ injection and 7 weeks of SGLT2i (low and high dose) treatment. Representative immunohistochemical stainings of pancreatic tissue for glucagon (**A**), insulin (**B**) and ELISA (**C**) or RIA (**D**) based determination of insulin serum levels. The data are the means ±SEM of 3–4 (**A,B**), 3–5 (**C**) or 9–11 (**D**) animals/group. *, p<0.05 vs. control and ^#^, p<0.05 vs. STZ-injected group.

During the treatment period 3 (STZ), 1 (SGLT2i low dose) and 1 (SGLT2i high dose) rats died. The weight gain was significantly decreased in the STZ-treated diabetic rats and not significantly affected by a low dose but increased by a high dose SGLT2i therapy. Diabetic animals had higher triglyceride and total cholesterol levels compared to controls and to diabetic animals treated with SGLTi. Blood glucose was higher in all STZ-treated groups (approximately 4-fold as compared to the control group) when measured 3 days after STZ injection, clearly excluding any interference of the drug therapy (which was started 7 days after STZ injection) with the induction of diabetes. Blood glucose levels were determined 8 weeks after STZ injection and the untreated STZ rats showed an almost 5-fold increase in this parameter, which was dose-dependently decreased by SGLT2i therapy in non-fasting rats. In fasting rats, blood glucose levels were increased almost 4-fold in the untreated STZ rats and were reduced by low and high dose SGLT2i therapy to near control levels. The parameter for long-term glycemic conditions, HbA1c, was increased more than 4-fold by STZ injection and was dose-dependently decreased by SGLT2i therapy. Trichrome staining of pancreatic tissue revealed morphological changes in the islet cells in untreated diabetic rats (Figure S1 in File S1). Glucagon production as detected by immunohistochemical staining of pancreatic tissue, showed no obvious change in control, diabetic and SGLT2i treated groups (if at all a moderate increase in staining in the STZ group) (Figure 1A). In contrast, immunohistochemical staining for insulin showed a clear decrease in the STZ group as compared to the controls with only minor part of the islets producing any insulin at all (not shown) and even in these “active” islets only few β-cells were producing insulin. Low dose SGLT2i therapy showed some improvement of islet insulin production which was clearly increased by high dose SGLT2i therapy (Figure 1B). In accordance with these observations, the serum insulin levels were significantly decreased to background concentrations (measured both by ELISA and RIA) in the STZ group and dose-dependently normalized by SGLT2i therapy (Figure 1C and D). Interferon-γ levels showed no significant changes (Table 2).

**Table 2:** Weight gain, and blood and serum parameters in controls and diabetic rats.

	In vivo treatment group			
Parameter^a^	Ctr	STZ	STZ+low SGLT2i	STZ+high SGLT2i
Weight gain [g]	137±10 (n = 12)	27±19 (n = 8)*	43±19 (n = 9)*	97±14 (n = 10)^#§^
Blood glucose [mg/dl], 3d post STZ, non-fasting, before SGLT2i treatment	120±6 (n = 7)	441±22 (n = 9)*	457±22 (n = 11)*	447±26 (n = 11)*
Blood glucose [mg/dl], 8w post STZ, non-fasting	132±8 (n = 11)	509±20 (n = 9)* ^#^	336±49 (n = 10)* ^#^	230±30(n = 10)* ^#§^
Blood glucose [mg/dl], 8w post STZ, fasting	117±4 (n = 11)	395±42 (n = 9)*	185±15 (n = 10)^#^	168±19 (n = 10)^#^
HbA1c [mmol/mol]	24±2 (n = 10)	99±12 (n = 8)*	68±8 (n = 8)* ^#^	40±6 (n = 8)* ^#§^
HbA1c [%]	4.3±2.3	11.2±3.2	8.4±2.9	5.8±2.7
Cholesterol [mg/dl], ELISA	83±6 (n = 9)	105±8 (n = 7)*	93±4(n = 7)	83±5 (n = 7)^#^
Cholesterol [mg/dl], HF5^b^	102±5(n = 11)	125±4 (n = 9)*	108±4 (n = 9)^#^	105±3 (n = 9)^#^
Triglycerides [mg/dl]	87±7(n = 7)	273±79 (n = 5)*	178±31 (n = 6)	95±15 (n = 7)^#^
Interferon-γ [pg/dl]	n.d.(n = 5)	6±3 (n = 4)	2±2 (n = 4)	n.d. (n = 3)

a Weight gain was calculated from the difference of values prior and 8 weeks post STZ injection. Blood glucose was determined three days after STZ injection without SGLT2i treatment; fasting and non-fasting blood glucose as well as HbA1c levels were measured 8 weeks post STZ injection. The data are the means ± SEM of the indicated number of animals/group; n.d. means not detectable.

*, p<0.05 vs. control and ^#^, p<0.05 vs. STZ-injected and ^§^, p<0.05 vs. low dose SGLT2i treated.

b Separation of HDL and LDL using HF5 (Hollow Fiber Flow Field Flow Fractionation).

### Vascular parameters

**Figure 2:**
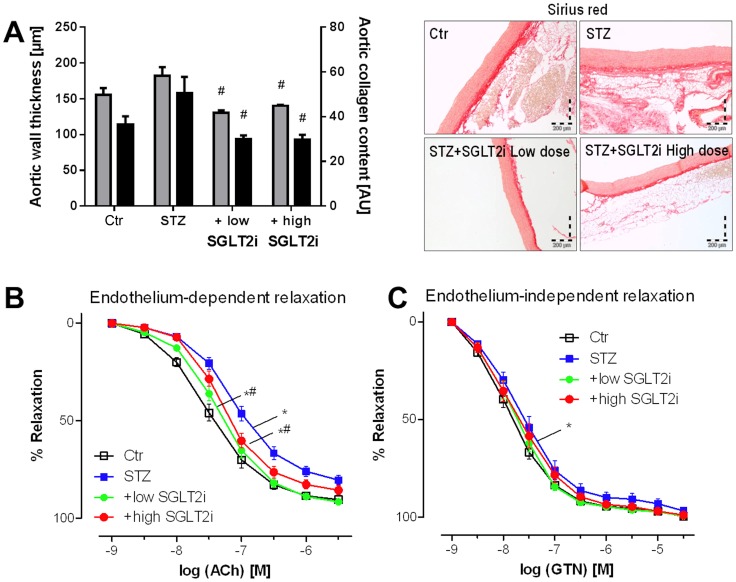
Effects of SGLT2i treatment on vascular parameters in diabetic rats. Microscopic determination of wall thickness (grey) and collagen content (black) by sirius red staining of paraffinated aortic sections (**A**). Representative microscope images are shown along with the densitometric quantification. Effects of SGLT2i therapy on endothelium-dependent and independent vascular relaxation by the vasodilators acetylcholine (ACh, **B**) and nitroglycerin (GTN, **C**), respectively. Data are the means±SEM from 6–7 (**A**) and 9–12 (**B,C**) animals/group. Each single value for an animal corresponds to the means of 4 individual aortic rings from this animal. *, p<0.05 vs. control and ^#^, p<0.05 vs. STZ-injected group. For the vascular function data the significance levels were determined by two-way-ANOVA and significances for the entire curves are indicated when at least one compared concentration condition showed significant differences. Vasodilator potency (EC_50_, pD_2_) and efficacy (max. relaxation) were also calculated and subjected to statistical analysis using one-way-ANOVA. The data and results are presented in Table 1.

Histological staining for collagen (sirius red) and aortic wall (media) thickness revealed no significant increase in thickening (remodeling) in the STZ group but substantial reduction of these parameters by both doses of SGLT2i (Figure 2A). STZ injection caused endothelial dysfunction (acetylcholine [ACh]-dependent relaxation), which was improved by both SGLT2i doses (Figure 2B). The effects on endothelium-independent (nitroglycerin [GTN]) relaxation were less pronounced (Figure 2C). Vasodilator potency (EC_50_, pD_2_) and efficacy (max. relaxation) are presented in Table 1. KCl-induced vasoconstriction did not change in any group but the phenylephrine-induced constriction was increased in almost all STZ groups and interestingly not corrected by SGLT2i therapy (Figure S2 in File S1).

### Oxidative stress parameters

**Figure 3:**
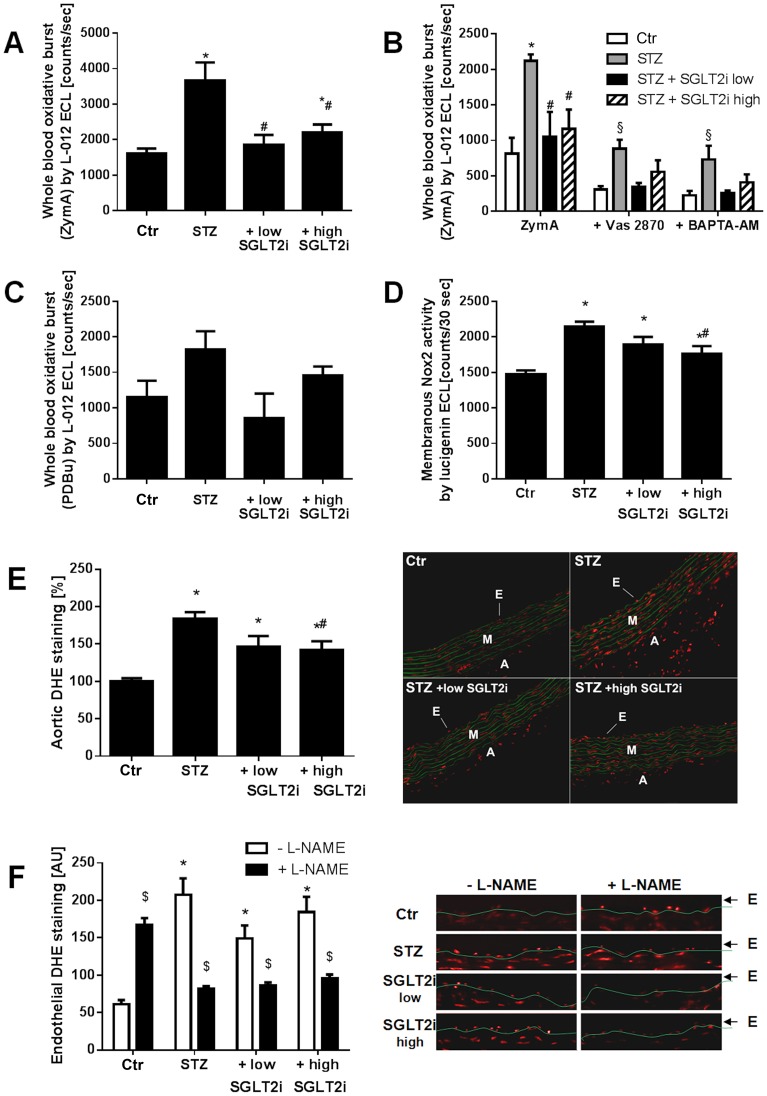
Effects of SGLT2i treatment on oxidative stress parameters in diabetic rats. Leukocyte-derived ROS (oxidative burst) in whole blood at 30 min upon zymosan A (**A**) stimulation along with the effects of the NADPH oxidase isoform 2 (Nox2) inhibitor VAS2870 and the intracellular calcium chelator BAPTA-AM (**B**) and upon PDBu stimulation (**C**). **(D)** Quantification of cardiac NADPH oxidase activity in membrane preparations by lucigenin (5 µM)-derived chemiluminescence. Dihydroethidium (DHE, 1 µM)-fluorescence microtopography was used to assess the effects of SGLT2i treatment on vascular (**E**) and endothelial (**F**) ROS production with and without incubation with the eNOS inhibitor L-NAME. Representative microscope images are shown along with the densitometric quantification. Red fluorescence indicates ROS formation whereas green fluorescence represents basal laminae autofluorescence. Data are the means±SEM from 6–7 (**A,B**), 5 (**C**), 7–8 (**D**), 9 (**E**) or 5–6 (**F**) animals/group. *, p<0.05 vs. control and ^#^, p<0.05 vs. STZ-injected and ^§^, p<0.05 vs. low dose SGLT2i treated and ^$^, p<0.05 vs. w/o L-NAME.

Oxidative burst in whole blood in response to zymosan A was increased in the diabetic rats and normalized by both SGLT2i doses and a similar pattern was observed in response to the synthetic phorbol ester analogue PDBu (mainly reflecting leukocyte NADPH oxidase activity) without displaying significant changes (Figure 3A and C). The signal in the STZ group was largely suppressed by the NADPH oxidase inhibitor VAS2870 (highest affinity for Nox2 isoform) and the intracellular calcium chelator BAPTA-AM, indicating the involvement of the phagocytic NADPH oxidase and [Ca^2+^]_i_ in this process (Figure 3B). NADPH oxidase activity from heart membranes was increased in the STZ group and dose-dependently inhibited by SGLT2i therapy (Figure 3D). In line with this, activity of the redox-sensitive enzyme mitochondrial aldehyde dehydrogenase (ALDH-2) was decreased in diabetic rats but not significantly impaired under SGLT2i therapy (Figure S3 in File S1).

Aortic reactive oxygen species formation was increased in the STZ group and reduced by high dose of SGLT2i, although still increased as compared to the control (Figure 3E). An almost similar pattern was observed for endothelial dihydroethidium staining of aortic cryo-sections, however, without significant reduction in the endothelial dihydroethidium fluorescence signal by either SGLT2i dose (Figure 3F). Experiments with the eNOS inhibitor L-NAME reduced the superoxide signal in the endothelial cell layer of vessels from the STZ group, compatible with eNOS uncoupling. SGLT2i treatment showed a mixed effect suggesting that eNOS uncoupling is not completely prevented. The dihydroethidium staining signal was sensitive to low concentration SOD treatment and high concentration catalase pre-incubation (Figure S4 in File S1).

### Protein expression

**Figure 4:**
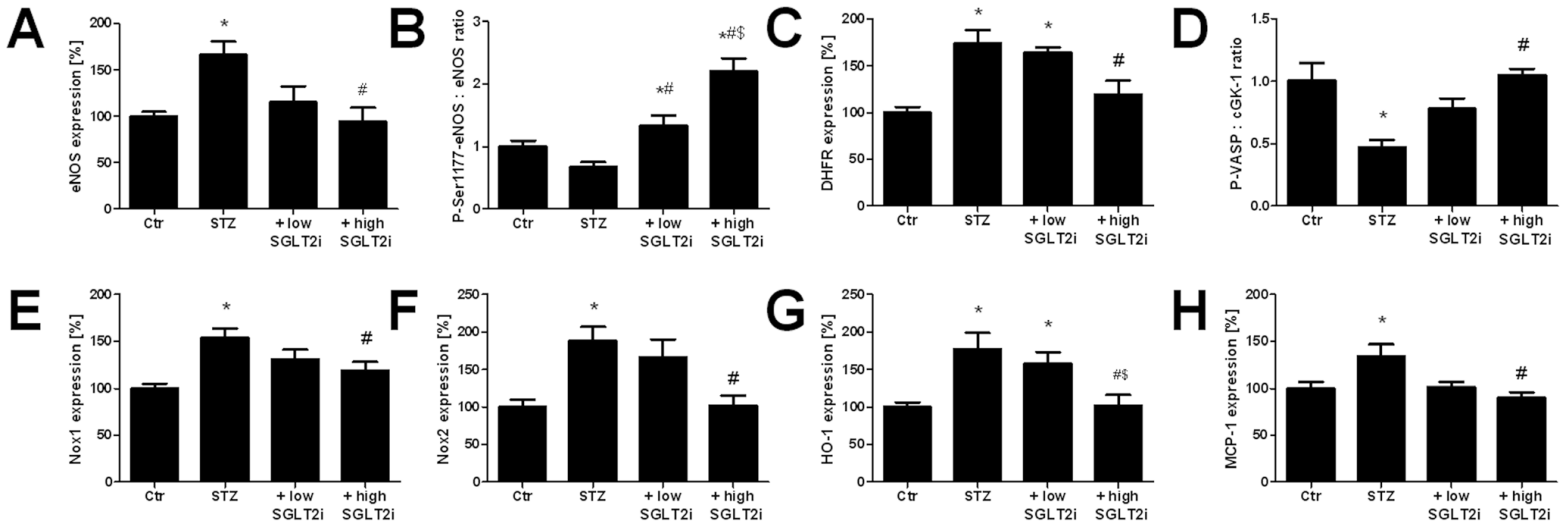
Effects of SGLT2i treatment on aortic protein expression of the NO/cGMP signaling cascade as well as oxidative stress and inflammatory pathways in diabetic rats. Expression of endothelial nitric oxide synthase (eNOS, **A**), serine1177 phosphorylated eNOS (**B**), dihydrofolate reductase (DHFR, **C**), ratio of cGK-I and serine239 phosphorylated VASP (**D**) were assessed by Western blotting analysis and specific antibodies. Expression of NADPH oxidases Nox1 (**E**) and Nox2 (**F**), heme oxygenase-1 (HO-1) (**G**) and monocyte-chemoattractant-protein-1 (MCP-1 or CCL-2, **H**) were assessed by Western blotting analysis and specific antibodies. Representative blots for all proteins are shown in supplemental Figure S6 in File S1. The data are expressed as % of control and are the means ± SEM from 8–9 (**A**), 5–6 (**B**), 7 (**C**), 4 (**D**), 6–7 (**E**), 7–9 (**F**), 7–9 (**G**) and 4–6 (**H**) animals/group. *, p<0.05 vs. control and ^#^, p<0.05 vs. STZ-injected and ^$^, p<0.05 vs. low dose SGLT2i treated.

We have shown previously that eNOS and dihydrofolate reductase (DHFR) expression was increased in aorta from diabetic animals suggesting compensatory up-regulation of these enzymes due to the dysfunction/uncoupling of eNOS and oxidative depletion of its co-factor tetrahydrobiopterin (BH_4_) with subsequent induction of dihydrofolate reductase, the BH_2_ recycling enzyme. STZ treatment significantly increased eNOS and dihydrofolate reductase expression and SGLT2i therapy decreased it dose-dependently suggesting that SGLT2i therapy normalized these adverse effects of hyperglycemia, thereby preventing the compensatory up-regulation of these enzymes (Figure 4 A and C). It should be noted that eNOS up-regulation per se is a beneficial event but if overexpressed in an oxidized environment (with depleted BH_4_ levels or in S-glutathionylated state) the enzyme might be uncoupled and might further aggravate the phenotype. Phosphorylation of eNOS at Ser1177, which increases eNOS activity, was impaired in STZ-treated rats and dose-dependently improved with SGLT2i therapy (Figure 4B). The expression of cGMP-dependent kinase was slightly increased in the STZ group and reduced to control levels by high dose SGLT2i therapy (Figure 4D, Figure S6D in File S1). Likewise, the phosphorylation of VASP at Ser239 (as a read-out of activation of the cGMP-dependent kinase) was decreased in the STZ-treated rats and normalized by high dose SGLT2i therapy (Figure 4D, Figure S6D in File S1). As a consequence, the ratio of P-VASP/cGK-I was significantly decreased in diabetic rats and improved dose-dependently by SGLT2i therapy with no significant impairment as compared to controls under low dose SGLT2i therapy and significant improvement as compared to STZ group under high dose SGLT2i therapy (Figure 4D). The expression of NADPH oxidase isoforms Nox1 and Nox2 was increased in diabetes and significantly reduced by high dose SGLT2i therapy (Figure 4E and F). As a general stress response, the antioxidant enzyme heme oxygenase-1 (HO-1) was up-regulated in diabetic rats without therapy and significantly reduced by high dose SGLT2i therapy (Figure 4G). The inflammatory protein monocyte chemoattractant protein-1 (MCP-1 or CCL-2) was up-regulated in untreated STZ rats, and normalized by high dose SGLT2i therapy (Figure 4H).

### AGE/RAGE signaling

**Figure 5:**
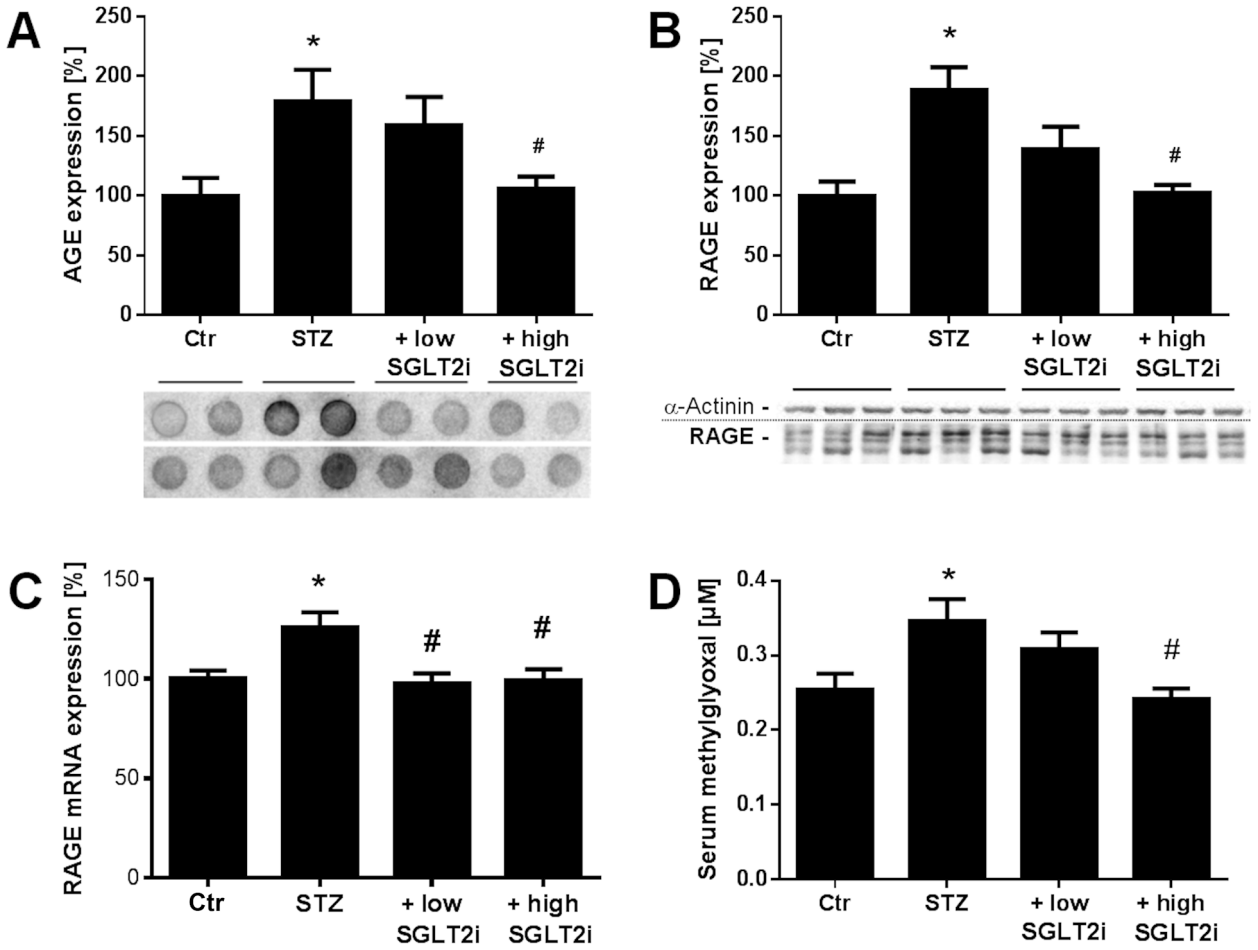
Effects of SGLT2i treatment on AGE/RAGE signaling in diabetic rats. Quantification of AGE-positive proteins by dot blot analysis (**A**) and RAGE expression was assessed by Western blotting analysis with specific antibodies (**B**) and quantitative RT-PCR analysis (**C**). Representative blots are shown at the bottom of the densitometric quantifications. Serum methylglyoxal levels were assessed by HPLC-based quantification (**D**). Representative chromatograms are shown in supplemental Figure S7 in File S1. The data are expressed as % of control and are the means ±SEM from 7 (**A**), 6–7 (**B**) and 8–11 (**C,D**) animals/group. *, p<0.05 vs. control and ^#^, p<0.05 vs. STZ–injected.

Quantification of advanced glycation end products (AGE)-positive proteins by dot blot analysis (Figure 5A) and AGE signaling was assessed by expression levels of their receptor (RAGE) at mRNA and protein level (Figure 5B and C). Aortic RAGE protein expression and levels of AGE-positive proteins was increased in the STZ group and normalized by a high dose SGLT2i therapy. Aortic RAGE mRNA expression showed similar results. In agreement with this, serum levels of the AGE precursor methylglyoxal were significantly increased in the STZ group and improved by high dose SGLT2i therapy (Figure 5D).

### mRNA expression

**Figure 6:**
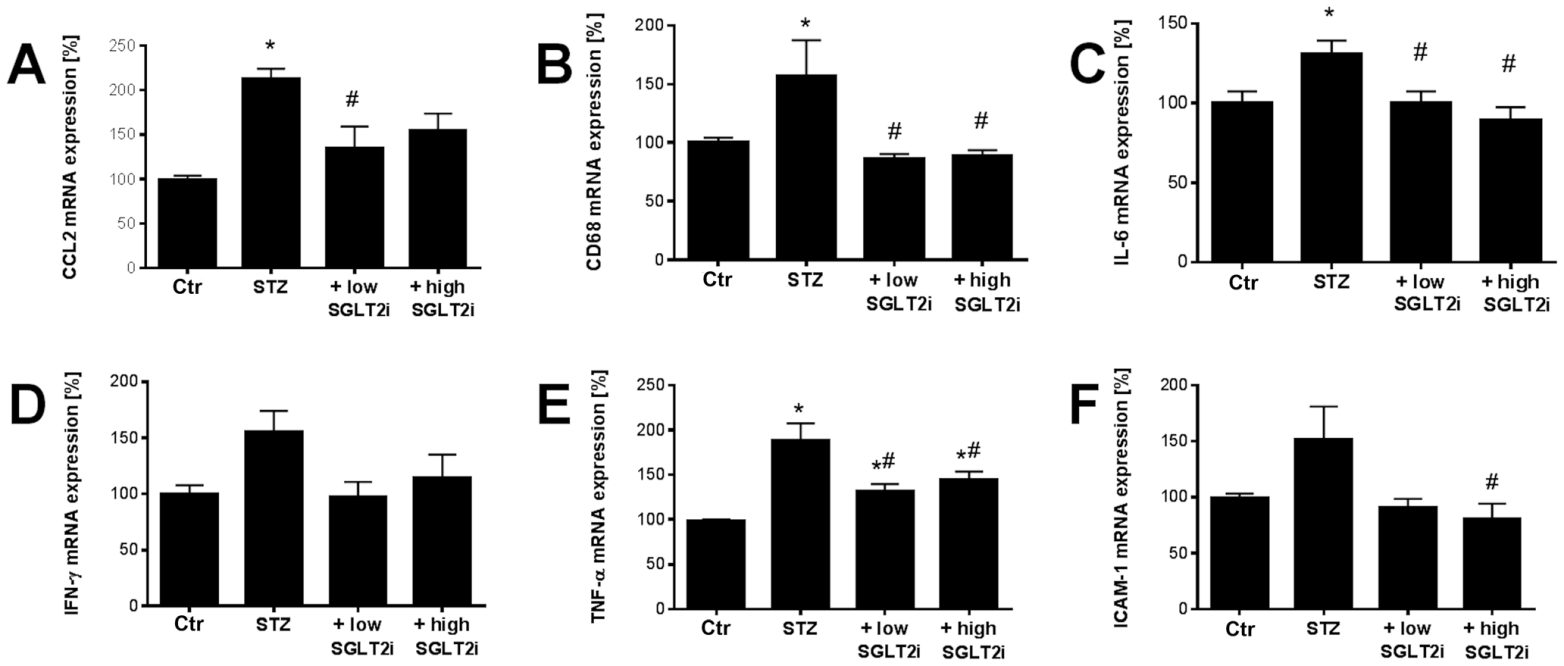
Effects of SGLT2i treatment on aortic mRNA expression of pro-inflammatory genes in diabetic rats. Expression of the monocyte chemoattractant protein-1 (CCL-2, **A**), the monocyte/macrophage-specific protein CD68 (**B**), the cytokine interleukin-6 (IL-6, **C**), the immune-signaling proteins interferon-γ (IFN-γ, **D**) and tumor necrosis factor-α (TNF-α, **E**) and the intercellular adhesion molecule-1 (ICAM-1, **F**) was assessed by quantitative RT-PCR. The data are expressed as % of control and are the means ±SEM from 7–9 (**A**–**C**), 9–10 (**D**–**E**) and 6–7 animals/group. *, p<0.05 vs. control and ^#^, p<0.05 vs. STZ-injected.

We also determined a number of inflammation markers by RT-PCR-based measurement of mRNA levels. The already mentioned monocyte chemoattractant protein-1 (MCP-1 or CCL-2, Figure 6A), the monocyte/macrophage-specific protein CD68 (Figure 6B) and the cytokine interleukin-6 (IL-6, Figure 6C) were up-regulated at the mRNA level in the aorta and significantly reduced by at least one SGLT2i dose. Likewise, the immune-signaling protein tumor necrosis factor-α (TNF-α, Figure 6E) was up-regulated in STZ-treated rats and reduced by both doses of SGLT2i, a pattern that was mirrored for interferon-γ (IFN-γ) and intercellular adhesion molecule-1 (ICAM-1) expression without reaching significant differences (Figure 6D and 6F). The general marker of inflammation, cyclooxygenase-2 (COX-2, Figure S5 in File S1) was increased in diabetic rats without treatment and normalized by both doses of SGLT2i.

## Discussion

**Figure 7:**
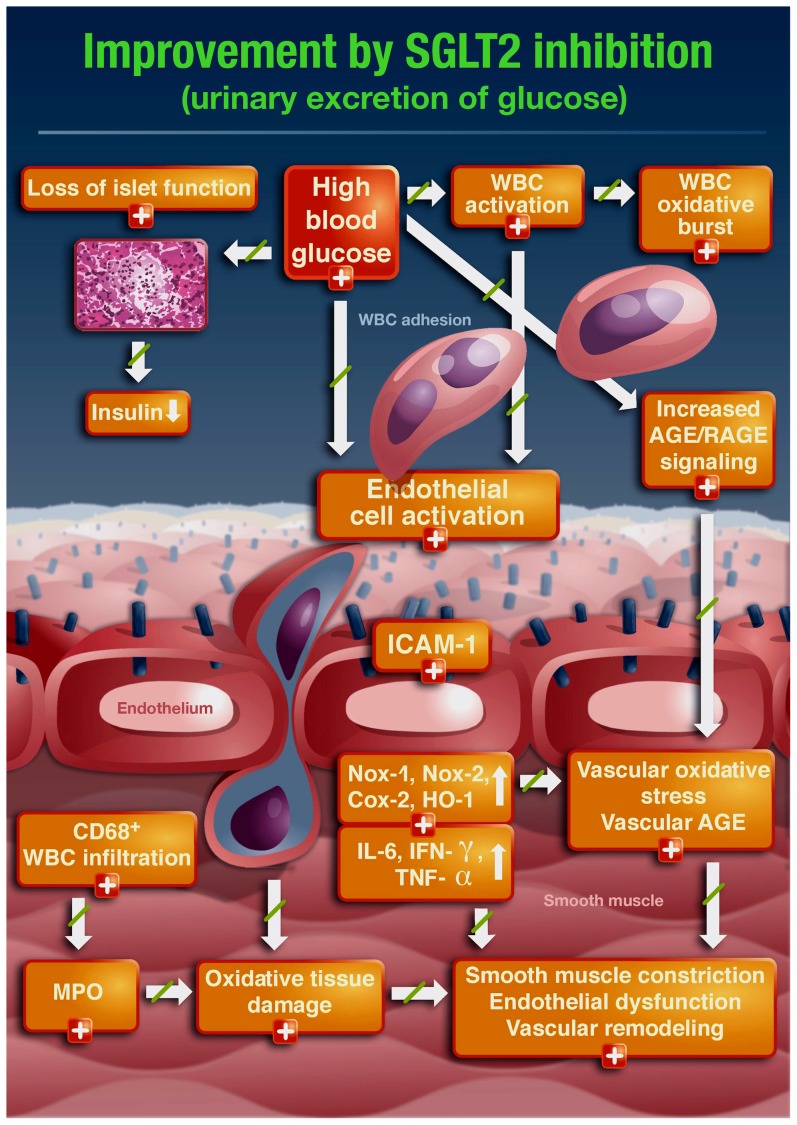
Summary of beneficial effects of SGLT2i treatment on diabetes induced vascular dysfunction and other adverse effects of hyperglycemia in rats.

With the present study we demonstrate that chronic treatment of STZ-treated rats with the SGLT2i empagliflozin prevents the development of endothelial dysfunction, oxidative stress, AGE/RAGE signaling and inflammation in a well characterized animal model of type I diabetes mellitus. These beneficial effects are mainly due to antioxidant and anti-inflammatory effects of this compound, such as an inhibition of the activity of NADPH oxidase and decreased serum levels of the AGE precursor methylglyoxal. These antioxidant and anti-inflammatory effects are likely due to glucose lowering effects. Glucose lowering is likely due to removal of glucose by the kidney via SGLT2 inhibition but may also be secondary to improved glucose utilization by restored insulin production and signaling, all of which prevent AGE formation, AGE/RAGE signaling, metabolic dysfunction, oxidative stress and impairment of vascular function. As expected, SGLT2i-dependent glucose lowering decreases cellular glucotoxicity and thereby contributes to the survival of pancreatic islet cells and preserves insulin production (Figure 7).

Diabetes is the most common cause of blindness, non-traumatic amputations and end-stage renal disease and the sixth most common cause of death. Yet only limited knowledge exists about the pathogenesis, the cardiovascular consequences and the prevention of this disease. Previous data suggests that the cardiovascular consequences of diabetes mellitus are secondary to oxidative stress [3], [6], [25], as previously shown for hypercholesterolemia, hypertension or established atherosclerosis. NADPH oxidases, mitochondria, xanthine oxidase, as well as uncoupled eNOS have been identified as major sources of these reactive oxygen and nitrogen species [4], [5], [33], [34]. Importantly, the efficacy of the current anti-diabetic treatments vanishes over time, leading to a progression of the disease [18]. This phenomenon is mostly explained by the reduction of the ß-cells' capacity to secrete insulin and by a desensitization of the insulin signalling pathways, while most of the treatments aim to induce insulin release or to improve insulin sensitivity. Mode of action of SGLT2 inhibitors is insulin independent, thus efficacy of empagliflozin should be preserved independently of the advancement of the ß-cell failure. Along these lines SGLT2 inhibitors have been demonstrated to be effective in diabetes mellitus type II [35] and are anticipated to be also effective in type 1 diabetic patients. The proof of concept that empagliflozin is effective in lowering glucose and sparing insulin has been recently demonstrated in a preclinical model of STZ-induced type 1 diabetes [22] but also in human studies [36], [37].

In contrast to the classical anti-diabetic therapies, SGLT2i treatment removes the glucose from the body and is thereby highly efficient in preventing so-called glucotoxicity, increased methylglyoxal levels, formation of AGE and induction of RAGE. Importantly, methylglyoxal has recently been demonstrated to play an essential role in the development and progression of diabetic neuropathy in diabetic mice and patients and therefore represents a target of special interest for pharmacological modulation [38]. Increased AGE/RAGE signaling in STZ rats has been established [39] and its contribution to increased oxidative stress and vascular complications under hyperglycemic conditions was identified [40]. In 2001, it was demonstrated that macrophages from gp91phox deficient mice responded less to AGE stimulation providing a direct link between AGE/RAGE signaling and NADPH oxidase expression/activity [12]. The same authors also demonstrated a connection between AGE/RAGE signaling and inflammation by increased VCAM-1 and tissue factor expression in human endothelial cells in response to AGE treatment. Increased tissue levels of macrophages were identified by MCP-1 and CD68 staining in STZ rats [41]. Increased TNF-α, IFN-γ expression as well as ROS producing enzymes in STZ rats were normalized by multiple antioxidant therapy [42]. Therefore, hyperglycemia via increased AGE/RAGE signaling leads to oxidative stress from NADPH oxidases, impaired NO/cGMP signaling and low-grade inflammation as supported by the present protein and mRNA expression data and oxidative stress measurements. All of these adverse effects were normalized by SGLT2i treatment.

In line with this, the burden of oxidative stress was decreased by SGLT2i at all levels (whole blood, aorta, heart) supporting the concept of a cross-talk between AGE/RAGE signaling and ROS formation in diabetes mellitus. The contribution of calcium signaling in this process was demonstrated by the inhibitory effect of intracellular calcium chelation in the present work and the overall importance of calcium for the interaction of mitochondrial ROS with the activation of NADPH oxidases (Nox1 and Nox2) was demonstrated recently [15]. Related to the oxidative stress concept, the redox regulated mitochondrial aldehyde dehydrogenase (ALDH-2) is not only important as a nitroglycerin bioactivating enzyme [43], [44] but also is an important antioxidant enzyme by degrading toxic aldehydes such as acetaldehyde, malondialdehyde and 4-hydroxynonenal [45], [46]. More recently it has been shown that ALDH-2 plays an important role for the reduction of myocardial damage during ischemia/reperfusion by reducing the infarct size [47] and conferring cardio-protection in an experimental diabetes/MI model [48]. Therefore, the inactivation of ALDH-2 in the STZ group substantially supports the cardio-toxic phenotype of hyperglycemia and improved activity of this redox-sensitive enzyme by a high dose SGLT2i therapy clearly points to cardio-protective and anti-oxidant properties of this drug.

### Limitations of the study

There are some limitations in the present study. We used not all animals for all assays. Therefore, to ensure that the treatment with STZ and other drugs was comparable among all rats, some parameters were measured in all of them (e.g. blood glucose, HbA1c, cholesterol, insulin by RIA, vascular function and oxidative stress (dihydroethidium staining)). The here observed increase in leukocyte activity (oxidative burst) in diabetic rats, which also nicely correlates with all other measured markers of inflammation, is at variance with previous reports indicating impaired oxidative burst in neutrophils [49] and monocytes [50] in diabetic individuals, which correlates with the impaired phagocytic clearance of bacterial pathogens in these individuals. The major limitation of the study is that STZ treatment kills only some part of the islet cells directly. The remaining β-cells probably die due to glucotoxicity within the first 2 weeks after the injection of STZ. This represents a clear difference as compared to human T1DM where all islet cells are lost due to autoimmune-driven killing of these cells. This limitation will complicate the translation of our findings to the clinical situation. It is also unclear, whether the present results from T1DM can be translated to T2DM, which is the much more frequent form of diabetes in humans. Therefore, our future studies will address whether 1) much later onset of SGLT2i therapy (6 weeks after STZ for weeks 7 and 8 after STZ) will improve the diabetic complications even when all islet cells have died and 2) early onset of SGLT2i therapy (only the first 2 weeks after STZ, w/o treatment for weeks 3-8 after STZ) will still have a beneficial effect by preserving islet cell function. Finally, similar studies on SGLT2i therapy in a T2DM model (e.g. ZDF rats) and assessment of parameters of diabetic complications at all levels are highly recommended.

### Conclusions and clinical implications

The results of the present study confirm previous findings that vascular dysfunction in the setting of diabetes is associated with increased vascular oxidative stress, AGE/RAGE signaling and low-grade inflammation (Figure 7). The clinical implications of the present findings originate from the complex interaction of redox and inflammatory pathways in cardiovascular pathogenesis [15], [16] requiring multi-targeted therapy to improve the disease progression at all levels. The presented data clearly shows that SGLT2i therapy leading to enhanced glucose excretion improves glucose-induced vascular dysfunction by reducing glucotoxicity, oxidative stress, low-grade inflammation and restoring insulin signaling, ultimately improving endothelial dysfunction as an important determinant of future cardiovascular events.

## Supporting Information

File S1 Online supplemental data.
(PDF)
