# Supplementary material for: The Sodium-Glucose Co-Transporter 2 Inhibitor Empagliflozin Improves Diabetes-Induced Vascular Dysfunction in the Streptozotocin Diabetes Rat Model by Interfering with Oxidative Stress and Glucotoxicity
Source: PLoS One. 2014 Nov 17;9(11):e112394. doi: 10.1371/journal.pone.0112394 (PMC4234367; doi:10.1371/journal.pone.0112394)
Supplement: File S1 — Online supplemental data. (PDF) [file pone.0112394.s001.pdf]

Online supplemental data

The sodium-glucose co-transporter 2 inhibitor empagliflozin improves diabetes-induced vascular dysfunction in the streptozotocin diabetes model by interfering with oxidative stress and glucotoxicity

Matthias Oelze[a]*, Swenja Kröller-Schön[a]*, Philipp Welschof[a], Thomas Jansen[a], Michael Hausding[a], Yuliya Mikhed[a], Paul Stamm[a], Michael Mader[a], Elena Zinßius[a], Saule Agdauletova[a], Anna Gottschlich[a], Sebastian Steven[a], Eberhard Schulz[a], Serge P. Bottari[b], Eric Mayoux[c], Thomas Münzel[a], and Andreas Daiber[a]

From the [a] 2nd Medical Clinic, Department of Cardiology, Medical Center of the Johannes Gutenberg University, Mainz, Germany, [b] Laboratory of Fundamental and Applied, Bioenergetics, INSERM U1055, Grenoble-Alpes Université et Pôle de Biologie, CHU, Grenoble, France, [c] Boehringer Ingelheim Pharma GmbH & Co. KG, Ingelheim, Germany.

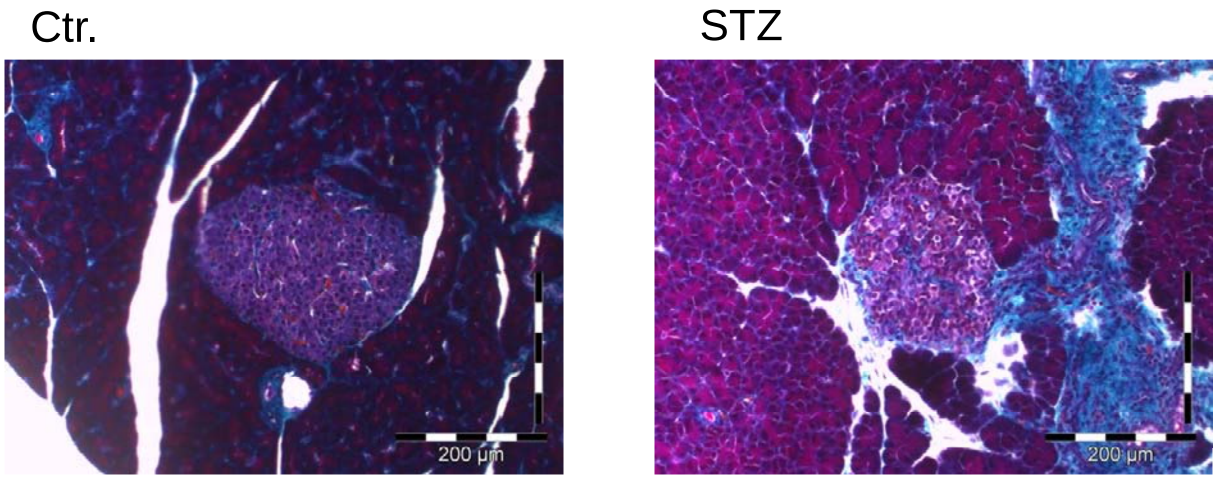

Figure S1. Histological trichrome staining of pancreatic tissue from control and diabetic rats. Increased sclerosis of islets in pancreas of diabetic rats. Representative microscopy images of 3-4 animals/group. Magnification 20x.

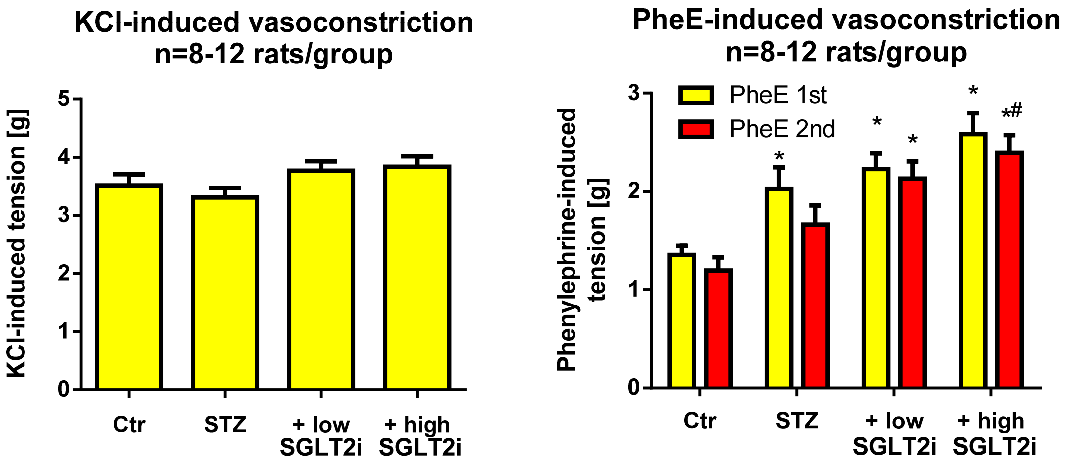

Figure S2. Effects of SGLT2i treatment on vascular parameters in diabetic rats. Effects of SGLT2i therapy on vasoconstriction of aortic ring segments by 80mM KCl (**left**) and 1µM phenylephrine (PheE, **right**), respectively. Data are the means±SEM from 8-12 animals/group. *, $p<0.05$ vs. control and #, $p<0.05$ vs. STZ-injected group.

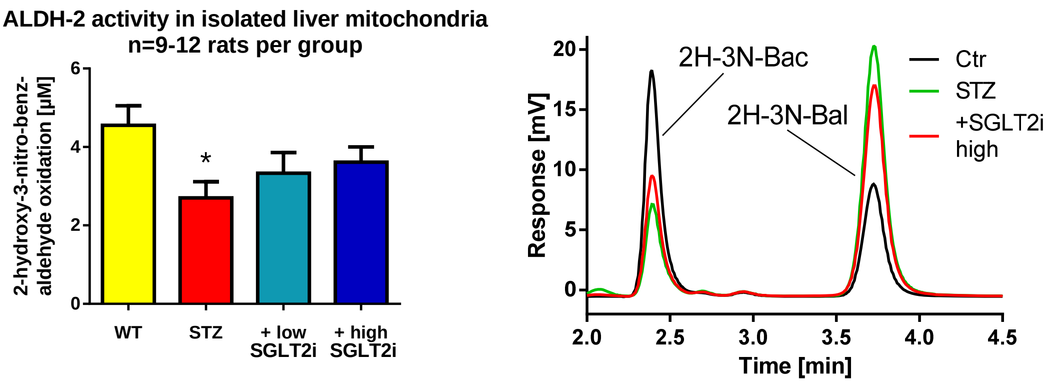

Figure S3. Effects of SGLT2i treatment on hepatic mitochondrial aldehyde dehydrogenase (ALDH-2) activity in diabetic rats. Effects of SGLT2i therapy on ALDH-2 activity in isolated liver mitochondria was measured by conversion of 2-hydroxy-3-nitro-benzaldehyde to its benzoic acid product. Representative chromatograms are shown along with the quantification. Data are the means±SEM from 8-12 animals/group. **, $p<0.01$ vs. control; *, $p<0.05$ vs. control and $^{\#}$, $p<0.05$ vs. STZ-injected group.

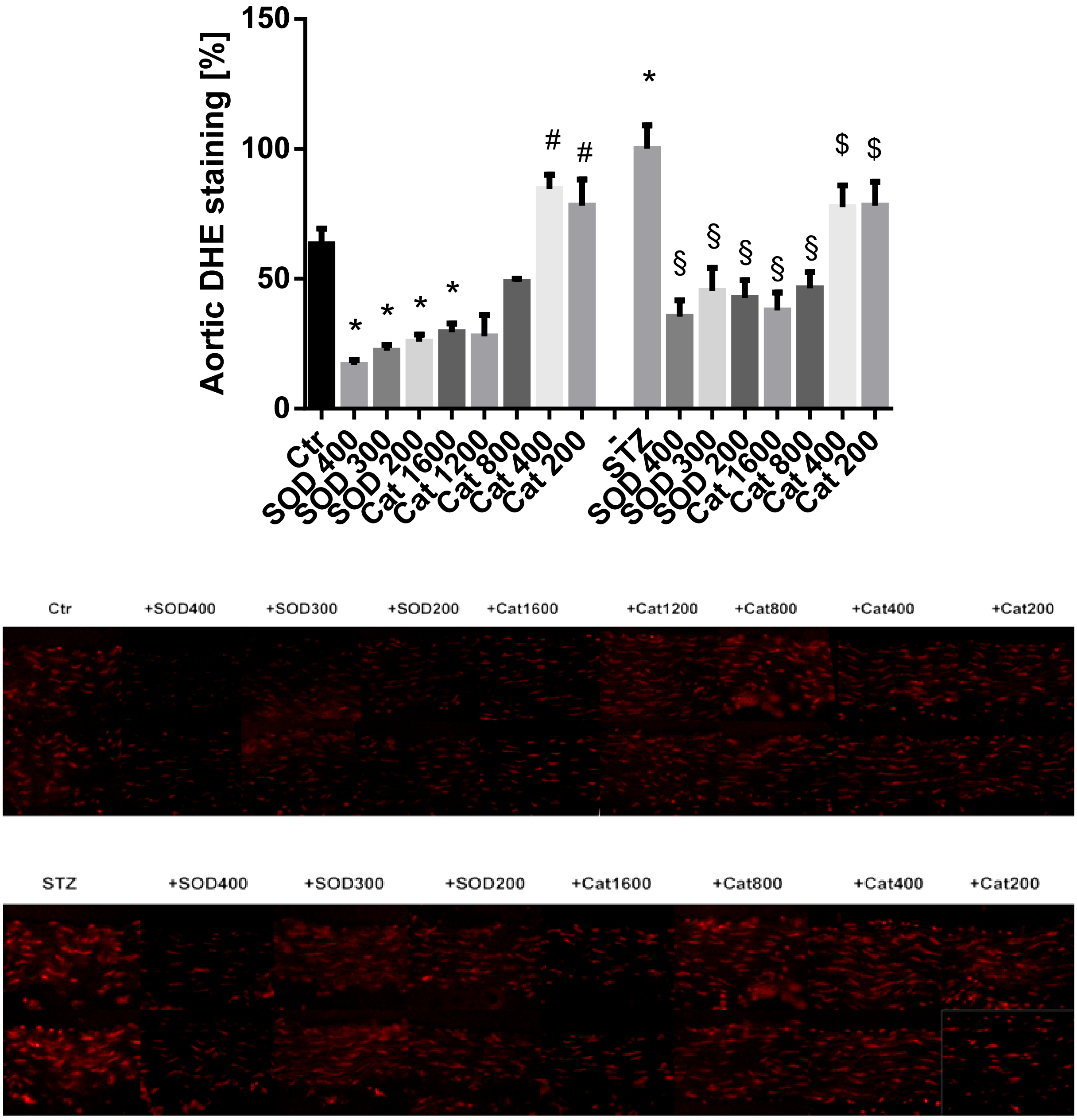

Figure S4. Effects of SOD and catalase on vascular ROS formation in control versus diabetic rats. DHE (1µM)-fluorescence microtopography was used to assess the effects of SOD and catalase pre-incubation (U/ml) ROS production. Representative microscope images are shown along with the densitometric quantification. Red fluorescence indicates ROS formation. Data are the means±SEM from 15 (Ctr and STZ) or 4-6 (all other groups) experiments. *, $p<0.05$ vs. control and §, $p<0.05$ vs. STZ and $^{\#}$, $p<0.05$ vs. Ctr-SOD400 and $^{\$}$, $p<0.05$ vs. STZ-SOD400.

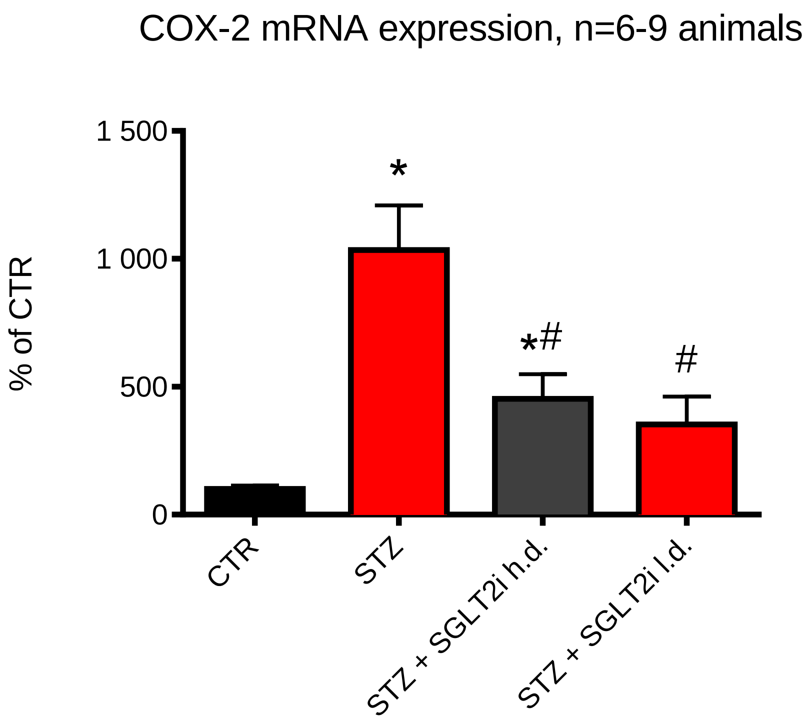

Figure S5. Effects of SGLT2i treatment on aortic mRNA expression of pro-inflammatory COX-2 gene in diabetic rats. mRNA expression of inducible cyclooxygenase (COX-2) was assessed by quantitative RT-PCR. The data are the means ± SEM from 6-9 animals/group. *, $p<0.05$ vs. control and #, $p<0.05$ vs. STZ-injected.

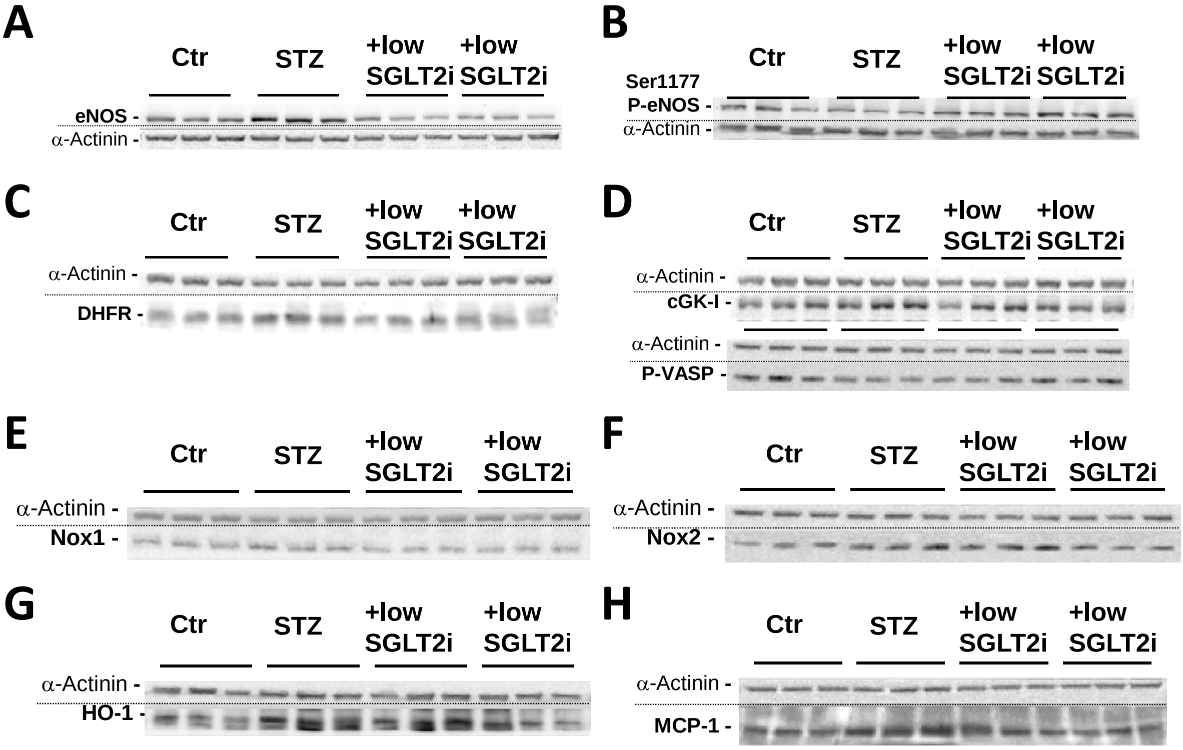

Figure S6. Effects of SGLT2i treatment on aortic protein expression of the NO/cGMP signaling cascade as well as oxidative stress and inflammatory pathways in diabetic rats. Representative Western blot stainings for expression of eNOS (**A**), serine1177 phosphorylated eNOS (**B**), DHFR (**C**), ratio of cGK-I and serin239 phosphorylated VASP (**D**), Nox1 (**E**), Nox2 (**F**), HO-1 (**G**) and MCP-1 (**H**) belonging to the densitometric quantification of these data presented in Fig. 4 of the main manuscript.

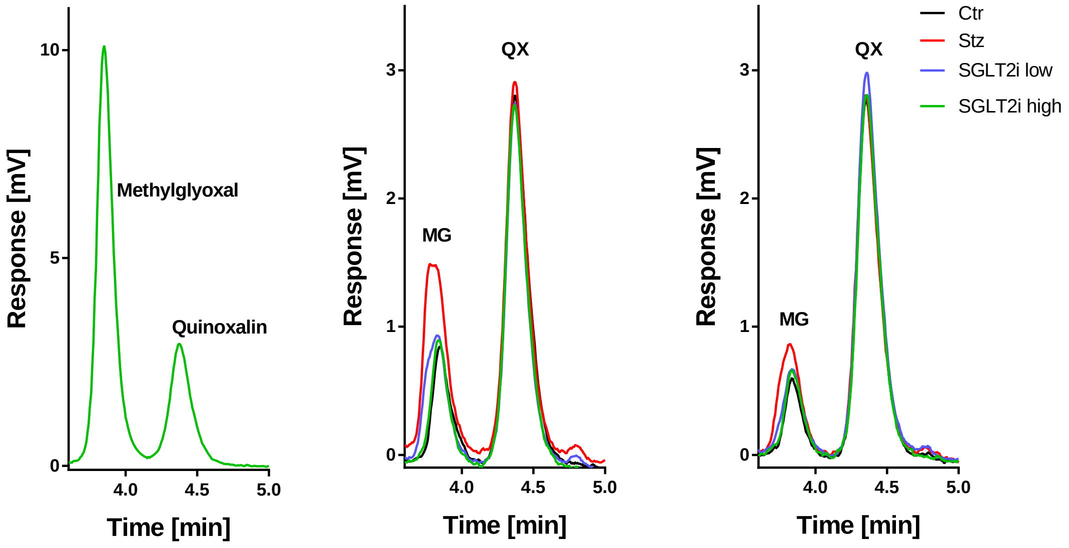

Figure S7. Effects of SGLT2i treatment on serum levels of the AGE precursor methylglyoxal in diabetic rats. Representative chromatograms of methylglyoxal (10 µM) and quinoxalin (10 µM) standards and two sets of serum samples from rats for the HPLC-based quantification of data presented in Fig. 5D of the main manuscript.

Extended Methods

Detection of serum cholesterol by Field-Flow Fractionation (FFF)

Total cholesterol in serum was also assessed by HF5 (Superon GmbH, Dernbach, Germany). Briefly, Field-Flow Fractionation (FFF) is a well-known family of separation methods that vary in the physical nature of the force field applied to generate separation [1]. Asymmetric Flow Field-Flow Fractionation (AF4) is the most popular type of FFF. It employs a flat or cyclindrical separation channel equipped with an ultrafiltration membrane and covers a wide separation range (1 nm - 1 μm). In HF5 the solvent is pumped through a porous fiber allowing a part of the flow to penetrate the wall, thus creating a cross flow which is perpendicular to the main solvent flow that has a parabolic profile and is directed to the fiber outlet. The combination of the two forces applied eventually results in the separation of the sample compound according to their respective diffusion coefficient (i.e. their hydrodynamic radius or molar mass, respectively). Like AF4, HF5 has a wide range of applications. It allows the separation of molecules in solution and particles in the same separation run. The separation takes place without the use of a stationary phase as in column chromatography. Consequently, there is less danger of sample adsorption or physical plugging of the separation channel. Another advantage of this technique is the low sample dilution due to the small channel volumes (< 100 μL) and low detector flow rates. The literature shows promising results for protein, nanoparticle, and even whole cell fractionation.

Extended references

1. Giddings JC (1993) Field-flow fractionation: analysis of macromolecular, colloidal, and particulate materials. Science 260: 1456-1465.
